# Management of Mesenteric Venous Ischaemia: A Case Series With Newer Approaches

**DOI:** 10.7759/cureus.25704

**Published:** 2022-06-07

**Authors:** Riadh Salem, Waseem Hameed, Radhakrishnan Ravikumar, Mookiah Bharathkumar, Jayachandran Devachandran, Kumarakrishnan Samraj

**Affiliations:** 1 General Surgery, Wexham Park Hospital, Slough, GBR; 2 Colorectal Surgery, Wexham Park Hospital, Slough, GBR; 3 Radiology, Apollo Hospitals, Chennai, IND; 4 Gastroenterology, Apollo Hospitals, Chennai, IND; 5 Intensive Care Unit, Apollo Hospitals, Chennai, IND; 6 Upper Gastrointestinal Surgery, Wexham Park Hospital, Slough, GBR

**Keywords:** mesenteric ischaemia, emergency & acute care, acute abdominal surgery, surgical thrombectomy (st), intestinal infarction, bowel ischemia

## Abstract

Mesenteric venous thrombosis is a rare condition that can result in morbid and sometimes fatal consequences. Conventional approaches have been to either resect and raise a stoma and/or anticoagulate. The disadvantage is that the conventional approaches do not address the underlying thrombus. This sometimes can lead to a downward spiral of worsening ischaemia culminating in further resections leading to loss of bowel length and subsequent short bowel syndrome. In this article, we present a case series that describes four possible approaches: (1) expectant management with anticoagulation, (2) resect, anti-coagulate, and reanastamose, (3) surgical thrombectomy (using Fogarty catheter), and (4) radiological thrombectomy. The technique along with criteria for different approaches are described

## Introduction

Mesenteric venous thrombosis is a rare condition that can result in morbid and sometimes fatal consequences [1]. Conventional approaches have been to either resect and raise a stoma and/or anticoagulate. Anticoagulation only prevents further formation of thrombosis. It doesn’t address the primary event.

In the absence of clinical or radiological signs of gangrene, conservative management with anticoagulation is recommended [2]. In the presence of necrosis, the dead gut is excised and a second look laparotomy is planned. In the interim, if signs of ischaemia persist, a transhepatic radiological approach should be considered prior to the second laparotomy. At the second-look laparotomy, if the bowel ends are viable they can be joined or a stoma raised depending on surgical discretion. We present surgical and radiological thrombectomy options, to rescue borderline bowel segments. The radiological procedure, if considered for ongoing ischaemia, would need to be done prior to second-look laparotomy. The decision for anastomosis or stoma is at the discretion of the operating surgeon. We present a case series of four patients with different treatment pathways.

## Case presentation

Patients and outcomes

The records and relevant images of the patients were reviewed retrospectively from the three hospitals (Dr. Kamakshi Memorial Hospital, Chennai, India; Apollo Specialty Hospital, Chennai, India; and Wexham Park Hospital, Slough, United Kingdom) where they presented as an emergency. Four patients with different treatment pathways are presented here. These patients were encountered over a period of nine years (2010-2019).

Patient 1

**Figure 1:**
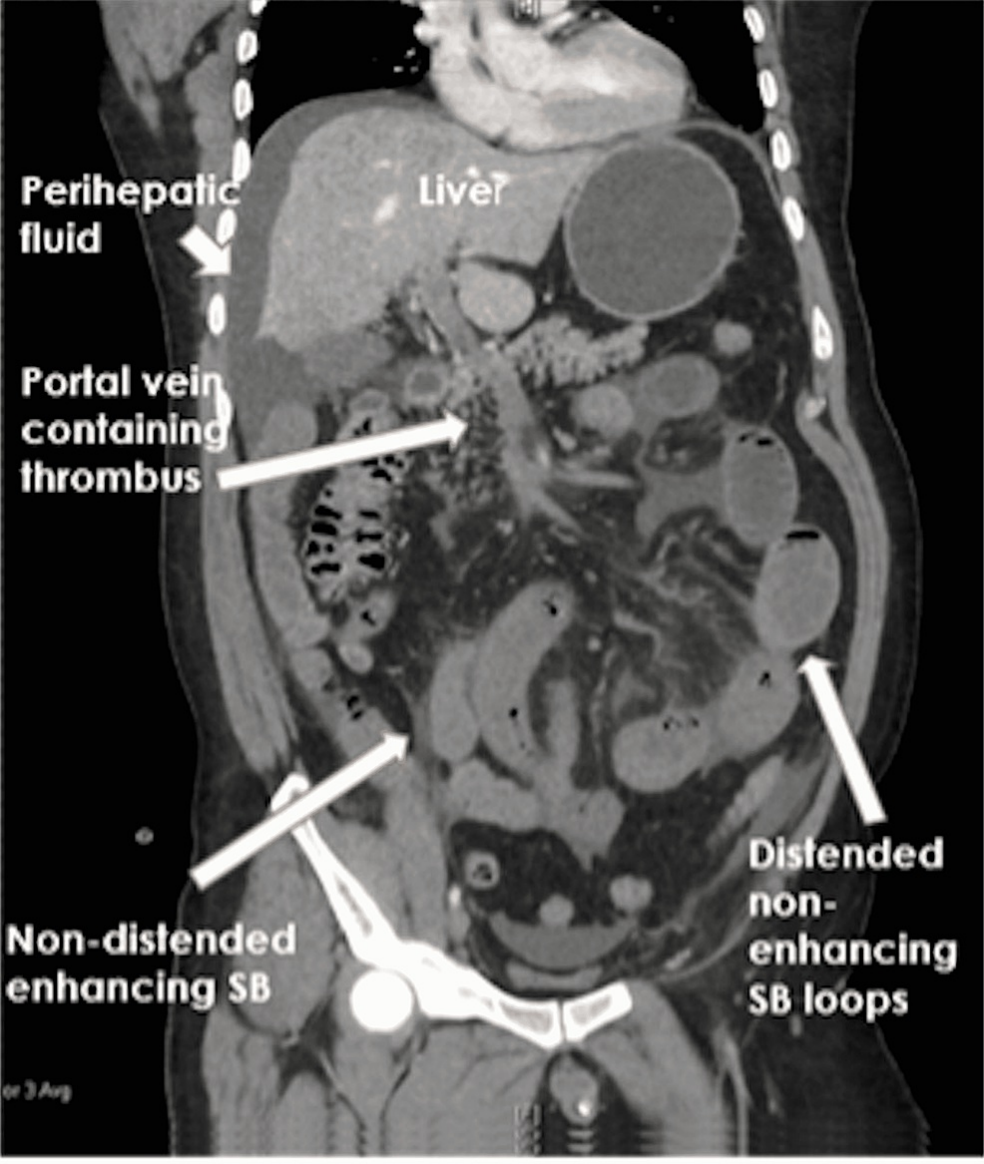
CT Abdomen showing venous thrombus and ischaemic bowels.

A 50-year-old gentleman presented with a four-day history of severe abdominal pain. He had recently returned from a long-haul flight. On examination, the abdomen showed generalised tenderness. There was no prior history of thrombosis or medical conditions. There was no history of any recent medical immobility. Contrast-enhanced CT of the abdomen revealed a superior mesenteric vein thrombus extending into the portal vein with secondary bowel ischaemia (Figure 1).

**Figure 2:**
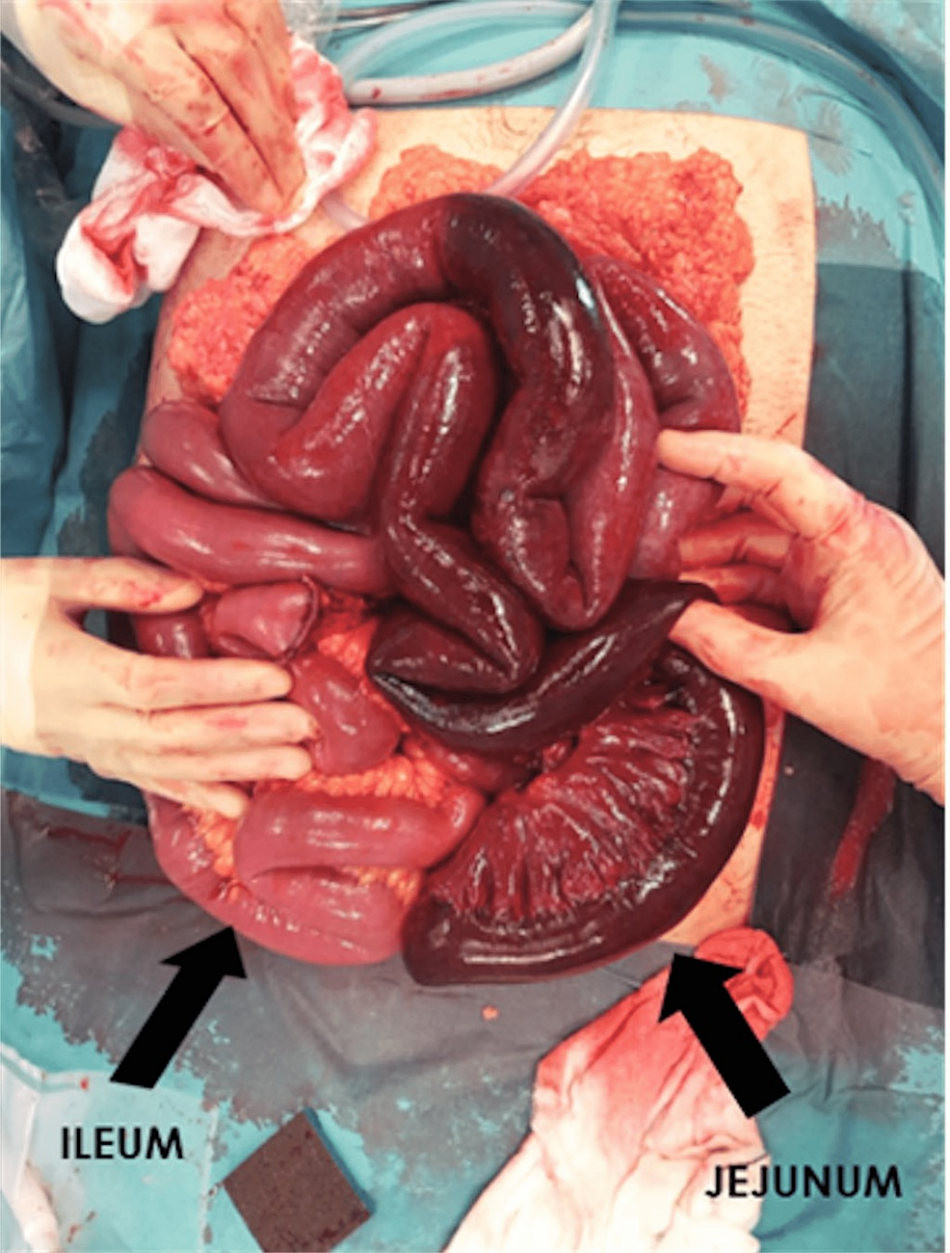
Intraoperative image showing infarcted small bowel.

At laparotomy, an ischaemic segment (Figure 2) measuring 130cm was resected (210cm beyond the duodenojejunal flexure). The stapled ends were left in situ. The abdomen was left open, and the patient was started on anticoagulation and managed in the intensive therapy unit (ITU) postoperatively. On the second-look laparotomy, the ends looked viable and were anastomosed.

**Figure 3:**
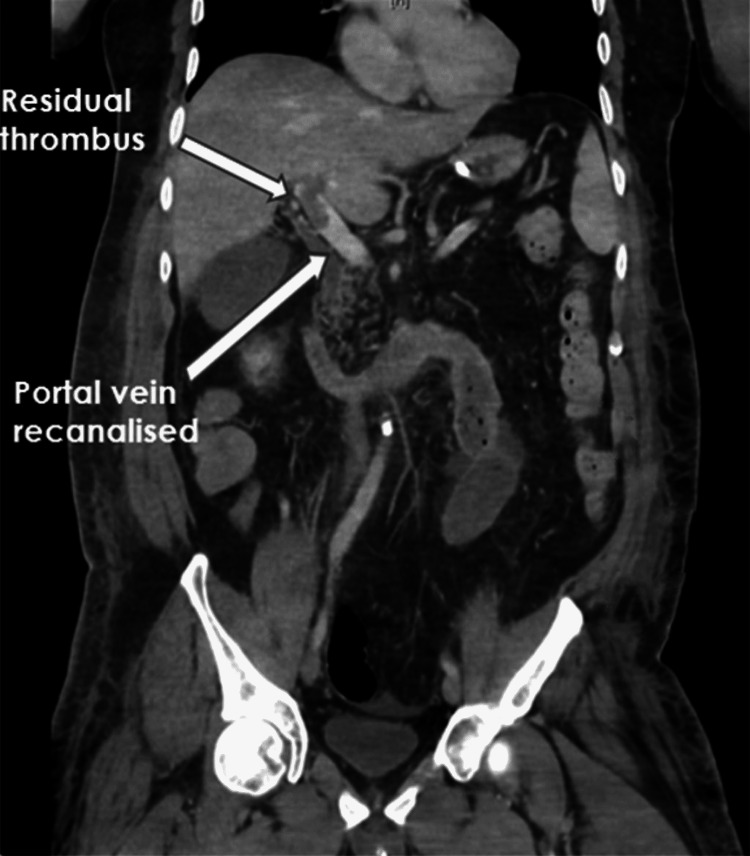
CT abdomen showing recanalisation of the portal vein.

A few days later, CT showed a recanalised portal vein with a small residual thrombus and healthy small bowel (Figure 3). 

The patient was seen by haematology team. Tests for antiphospholipid syndrome (APS), paroxysmal nocturnal haemoglobinuria (PNH), and multiple myeloma returned negative. Lifelong anticoagulation with warfarin, with a target international normalized ratio (INR) of 2-2.5 was advised. Upon discharge, an oesophagogastroduodeno-scopy and colonoscopy were normal. These were done to exclude any possible varices secondary to portal vein thrombosis. A year on he is on warfarin and asymptomatic.

Patient 2

A 30-year-old male patient with terminal ileal stricture due to inflammatory bowel disease underwent a laparoscopic limited right hemicolectomy due to failed medical measures. Six days after the operation he was readmitted with acute abdominal pain. An ultrasound showed mesenteric venous thrombosis. The pain settled after 24 hours, and due to cost issues, a CT scan was not done, though advised. Intravenous heparin was started and his pain slowly subsided. After 48 hours, this was changed to warfarin (target INR 2-2.5). An upper gastrointestinal (GI) endoscopy showed a single column of varix at the oesophagogastric junction. His liver function tests were normal both pre-and postoperatively. Subsequent ultrasound confirmed partial recanalization of the portal vein with signs of portal hypertension. He is on long-term warfarin as per haematology advice. 

Patient 3

**Figure 4:**
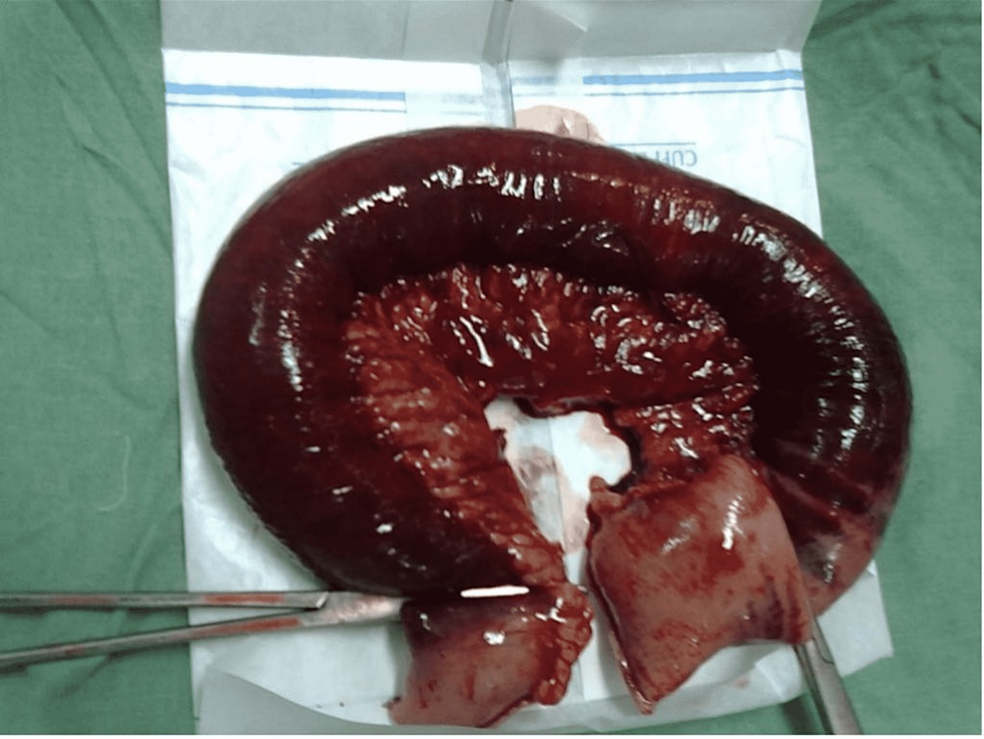
Showing two feet of the infarcted small bowel.

A 20-year-old male patient presented with sudden onset of severe abdominal pain. A CT scan showed dilated mid-small bowel loops. As the pain persisted with increasing abdominal guarding, a clinical decision for laparotomy was made. At surgery, approximately a two feet stretch of the terminal ileum was noted to be gangrenous (Figure 4). The rest of the small bowel looked dusky. Arterial pulsations were intact. Venous congestion was present. The non-viable segment was resected; the stapled ends were left in situ. Heparin was started. 

A relook laparotomy showed no further gangrene. However, the remaining bowel continued to look ischaemic. The bare mesenteric leaf from the previous excision was probed and a venous tributary cannulated with a Fogarty catheter. Thrombectomy was done by multiple passes (Video 1). 

**Video 1:** Showing the use of Fogarty catheter for venous thrombectomy

The bowel colour progressively improved. A completion contrast study showed a patent portal vein. As there was a marked improvement in the perfusion of the remaining small bowel, primary anastomosis was done. The patient recovered and was discharged on oral anticoagulants. Four weeks later he was readmitted with dyspnoea. CT images of the chest, abdomen, and pelvis showed pleural effusion with superior vena cava thrombosis (SVC). The effusion was drained and he recovered. A period without anticoagulation is necessary for thrombotic investigations. This was felt to be risky and hence the cause for this could not be safely investigated. In view of these events, lifelong anticoagulation was deemed necessary by the Haematologist.

Patient 4

**Figure 5:**
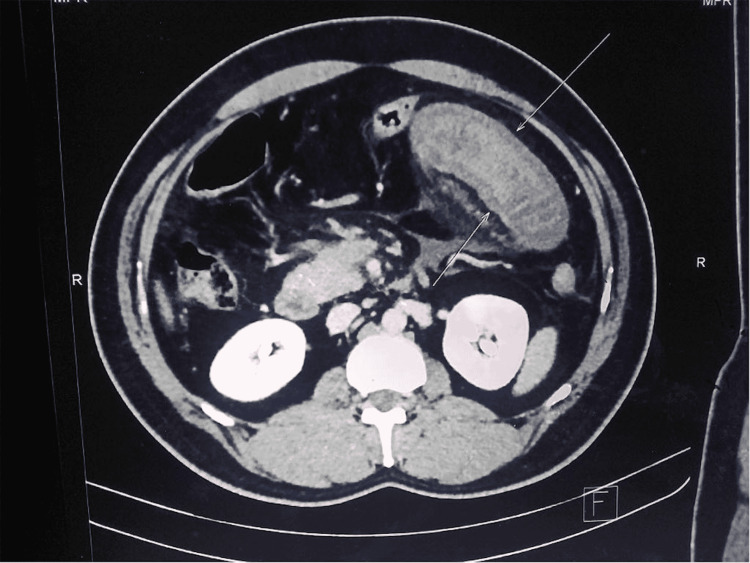
CT axial cross-section showing dilated small bowel loop.

**Figure 6:**
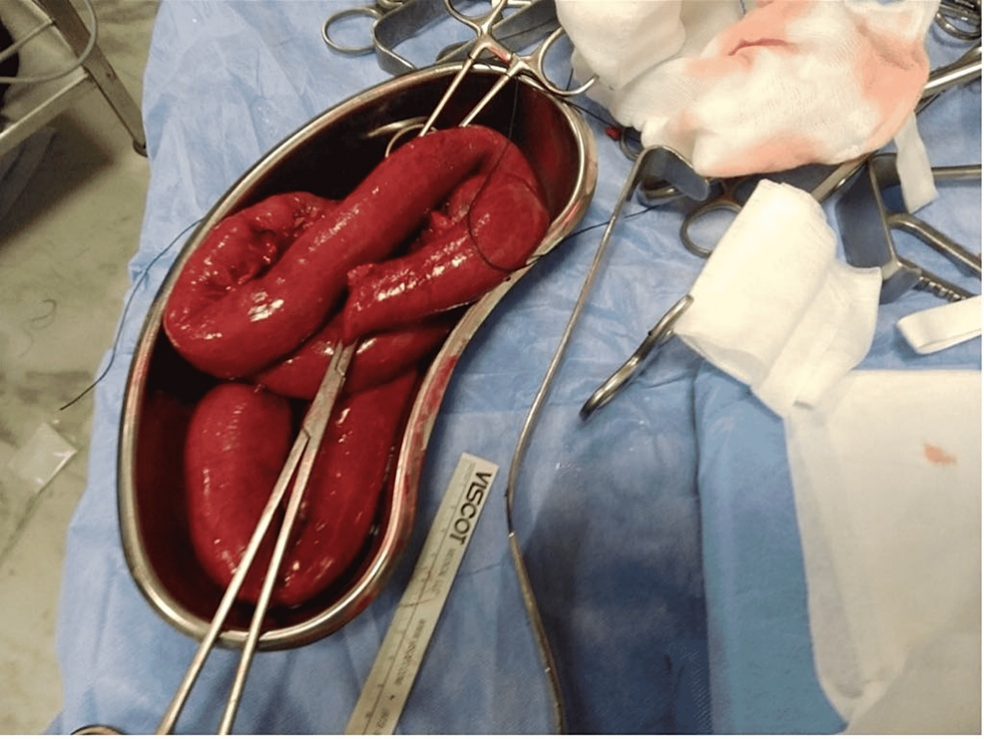
The resected segment of the ischaemic small bowel.

A 45-year-old male patient presented with acute abdominal pain along with tachycardia and hypotension. A CT showed a segment of jejunum that was ischaemic (Figure 5). At laparotomy, about 18 inches of jejunum was found to be infarcted (Figure 6). Arterial pulsations were intact. The non-viable segment was excised. The proximal segment was only four inches in length beyond duodeno-jejunal flexure. The ends were left stapled in situ.

In ITU, his condition improved but there was persistent acidosis. In view of this, radiology-guided thrombectomy was done. Transhepatic access to the portal system was obtained (Video 2). A catheter-guided clot aspiration was done. A completion study showed restitution of the portal vein. The catheter was left in situ. Urokinase was given as an infusion at a rate of 100,000 units per hour for five hours and later substituted by intravenous heparin (1000 units per hour and later adjusted by activated partial thromboplastin time {APTT}). On second-look laparotomy, the distal end needed further limited resection. A primary anastomosis was done. Three days later, the thrombectomy catheter was removed with coil embolisation of the tract. During substitution of heparin with enoxaparin, the patient developed HITS (heparin-induced thrombocytopenia) and pulmonary embolus. Enoxaparin was stopped and an alternative was commenced. After two years of follow-up, he is well on long-term anticoagulation.

**Video 2:** Showing transhepatic access.

## Discussion

Mesenteric venous thrombosis occurs in younger patients. The major cause is an underlying prothrombotic disorder [3]. The frequency and detection depend on the extent of investigations [4]. In our series, partly due to cost considerations and also the reluctance to stop anticoagulation for tests, our ability to investigate was limited in spite of appropriate referral to the haematologist. Two of our patients had a further episode of thrombus, which led the haematologist to suggest lifelong anticoagulation. In the past, most were classed as idiopathic. However, with advances in haematology and genetics, more patients are now identified to have an underlying prothrombotic aetiology [5]. The conventional approach to venous ischaemia has been to resect non-viable segments followed by anticoagulation [6]. The disadvantage of this is that it does not address the underlying thrombus. Sometimes there is a downward spiral of worsening ischaemia culminating in further resections leading to loss of bowel length and subsequent short bowel syndrome. Some patients end up needing a small bowel transplant in the event of intestinal failure [7]. Some are left with portal hypertension. The emergency often happens out of hours and resection is the usual preliminary approach. Consideration should be given to leaving the stapled ends inside in preparation for a relook laparotomy [8]. This often results in a more stable patient with time to explore further options. Anticoagulation prevents only thrombus propagation [9]. 

Our approach is to try addressing the thrombus at the second-look laparotomy in the event of continued ischaemia. Invasive approaches can be surgical or radiological. Where the involvement is distal, the bare mesentery at the excised area provides access to the inferior mesenteric vein and portal vein. Veins are stretchable and hence relatively bigger clot pieces can be extracted (as shown in Video 1). The venous system is under pressure. Coring multiple lumens through the thrombus achieve decompression and allows the body’s native fibrinolytic system to work faster, as the contact area is increased. Fogarty-assisted antegrade thrombus extraction has not been reported to our knowledge. It is easily done and within the skill set of most general surgeons. It results in minimal endothelial trauma as the entry point is ligated avoiding a venotomy suture line which can potentially trigger another thrombosis. Fogarty-assisted thrombectomy is only suitable for distal ileal involvement, as there is a possibility of access to the terminal part of the superior mesenteric vein. In the proximal small bowel, the venous radicles are thinner and longer. It is not possible to pass a Fogarty catheter through a tributary, without risking a tear in the main vessel. In this situation, a transhepatic radiological approach provides access (shown in Video 2). Clot aspiration by this route needs equipment and expertise which is available mostly in transplant centers, as was the case with the fourth patient. After the thrombus aspiration, it is of benefit to leave the catheter in situ for five days. The advantages are: (a) Access to administer urokinase directly into the portal system (these are however non-standard indications and relevant contraindications should be carefully considered); (b) Follow up infusion with heparin; (c) Contrast study assessment of possible recanalisation; and (d) Risk of bleeding is less as medications are given into portal system and systemic side effects are minimised.

During removal, care must be taken to embolise the hepatic track to minimise bleeding, as these patients need anticoagulation (Video 2). Due consideration should be given to a stoma where bowel continuity is not appropriate. However, in the two patients who underwent thrombectomy (surgical; radiological), the return of perfusion was so remarkable, that a primary anastomosis was done, with a good outcome. With our fourth patient, the proximal limb was too short for a stoma to be raised. The option at this stage was distal feeding jejunostomy and a proximal venting Foley from the small jejunal stump. This would have put the patient on a difficult nutritionally challenging pathway along with the formidable task of managing a high output proximal fistula. The return of perfusion and subsequent primary anastomosis obviated this. As discussed, further loss of bowel can be obviated by using these techniques. A decision flowchart is suggested in Appendix 1. 

## Conclusions

In summary, the options for managing mesenteric ischaemia depend on the viability of the bowel. In the absence of necrosis, conservative management with anticoagulation is usual practice (patient 2). In the presence of gangrene, resection is required. A second-look laparotomy is worth considering, as it gives time for anticoagulation and underlying ischaemia to stabilise. At second look laparotomy, if the bowel ends are viable, they can be joined (patient 1) or a stoma raised. If there are signs of continuing ischaemia, preparation should be made for either a preoperative radiological thrombectomy (if signs of persisting ischaemia) for proximal involvement (patient 4) or an on-table Fogarty guided thrombectomy (patient 3). A planned second-look laparotomy allows time for expertise to be organised for thrombectomy; surgical or radiological. The majority of patients in our case series are young and such patients can avoid lifelong morbidity from short bowel syndromes when these efforts are successful.

## Figures and Tables

**Figure 7:**
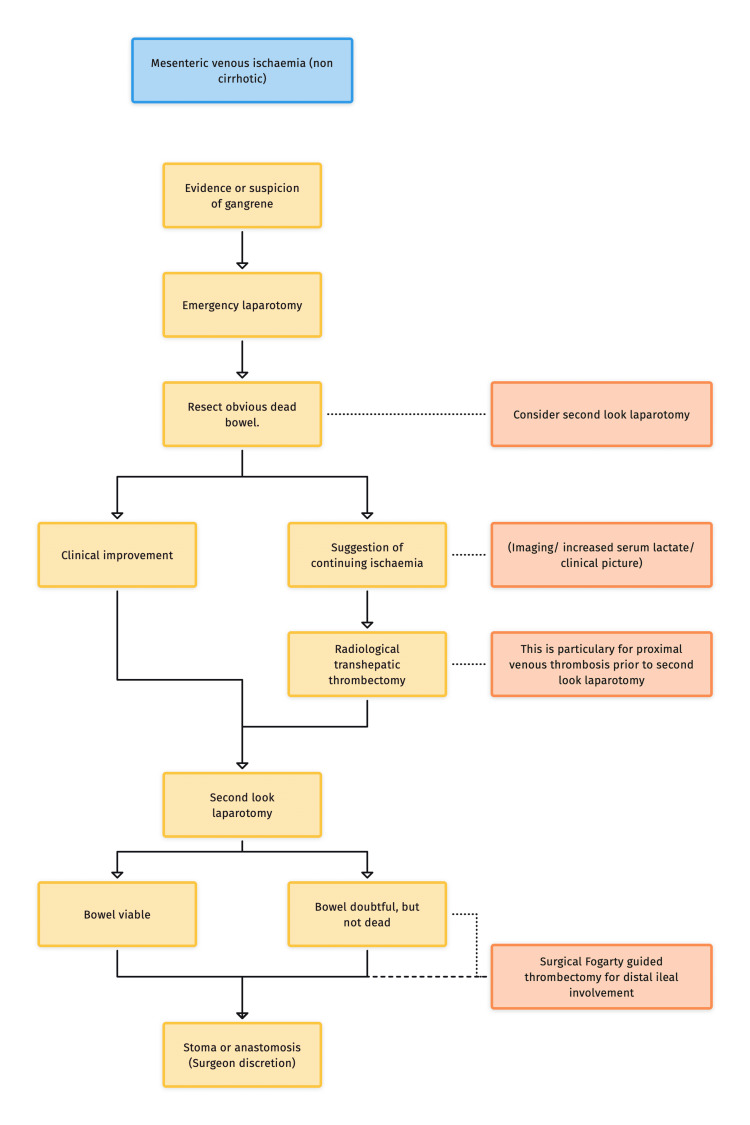
Management of mesenteric venous ischaemia
